# Unusual Chemotherapeutic Resistant Testicular Embryonal Germ Cell Tumor with Widespread Metastasis in a Case of Klinefelter Syndrome: A Case Report

**DOI:** 10.7759/cureus.7637

**Published:** 2020-04-11

**Authors:** Shah Huzaifa Feroz, Michael W Sistare, Jacob I Jabbour, Mohammad Masri, Carlos Dominguez

**Affiliations:** 1 General Surgery, Larkin Community Hospital, Miami, USA; 2 Surgical Oncology, Larkin Community Hospital, Miami, USA; 3 General Surgery, University of Miami, Miami, USA; 4 Medical Oncology, Larkin Community Hospital, Miami, USA

**Keywords:** embryonal cell carcinoma, klinefelter syndrome, testicular germ cell tumors, mediastinal germ cell tumor, retroperitoneal testicular germ cell tumors

## Abstract

Cryptorchidism is an undeniable risk factor for testicular germ cell tumors (TGCTs) and is also commonly associated with Klinefelter syndrome (KS) patients. Embryonal cell carcinoma usually shows strong expression of CD30 and OCT3/4, with patchy staining of PLAP1. Most patients with nonseminomatous GCTs (NSGCTs) can achieve total remission with proactive chemotherapy, and most can be cured. We present an extremely rare case of a testicular embryonal germ cell tumor that is atypical in its gene expression and response to chemotherapy treatment.

A 71-year-old male patient presented in July 2019 with abdominal pain of unknown duration, weight loss for one year, and recent history of altered bowel habits. His past medical history is significant for KS and congenital unilateral cryptorchidism. Physical examination yielded mild abdominal distention and bilateral inguinal lymphadenopathy. Imaging revealed a posterior mediastinal mass and large retroperitoneal masses. The above features, in addition to the history of KS and unilateral cryptorchidism, were highly suggestive of a testicular retroperitoneal germ cell tumor. Serologic studies revealed elevated lactate dehydrogenase (LDH) while other tumor markers were normal. Excisional biopsy of inguinal lymph nodes revealed poorly differentiated embryonal cell carcinoma with strong expression of SALL4, a rare expression of OCT 3/4, and the absence of expression of CD30 and placental alkaline phosphatase (PLAP). The patient was given four cycles of bleomycin, etoposide and platinum (BEP) chemotherapy, as is the standard chemotherapy regimen for these tumors, without any significant change in the size of the masses or lymph nodes.

Unfortunately, there are no specific guidelines when it comes to the management of KS patients with testicular GCTs (embryonal cell carcinoma) with aberrant histological markers and normal serum tumor markers. These findings in combination with chemotherapeutic resistance indicate a need for more specific treatment modalities and follow-up for unusual testicular embryonal GCTs in KS patients.

## Introduction

Klinefelter syndrome (KS) is characterized by a 47, XXY or a mosaic karyotype, and is responsible for hypergonadotropic hypogonadism. It affects approximately one in every 660 men, and <10% are diagnosed before puberty [1]. The classic presentation of KS is a tall-statured male with testicular atrophy (micro-orchidism), infertility, azoospermia, and gynecomastia. These men have a significantly increased risk for breast cancer (20x), and extragonadal (mediastinal) germ cell tumors (50x), but curiously these patients usually do not develop testicular tumors. Cryptorchidism is present in 27%-37% of KS subjects and is approximately six times more frequent than in the general male population [1]. Cryptorchidism is an established risk factor for testicular germ cell tumors (TGCTs) and about 10% of all cases of TGCT occur in men with a history of cryptorchidism. Seminomas have been commonly associated with cryptorchidism. Until now only a few cases of TGCT have been reported in KS with cryptorchidism, one of which being an intrapelvic seminoma [2-6]. Embryonal cell carcinoma usually shows strong expression of CD30 and OCT3/4, with patchy staining of PLAP1. Approximately 90% of patients with nonseminomatous GCTs (NSGCTs) can achieve a complete remission with aggressive chemotherapy, and most can be cured [7-8]. Here we present an extremely rare case of a KS patient with a metastatic testicular tumor. What makes this tumor especially rare is that the testicular tumor is an embryonal germ cell tumor. Furthermore, the embryonal GCT in our case is atypical in its gene expression and resistance to aggressive chemotherapy treatment.

## Case presentation

A 71-year-old male patient presented in July 2019 with abdominal pain of unknown duration, 107-pound weight loss over one year, and recent history of altered bowel habits. The abdominal pain was generalized, non-radiating, and not associated with alleviating or aggravating factors. The patient denied any nausea, vomiting, fevers, bone pain, or night sweats. The patient was also experiencing constipation with stools that were small and black. His past medical history is significant for KS, congenital unilateral cryptorchidism (right), a left atrophic testicle, cerebrovascular accident (CVA) in 2015, hypertension (HTN), chronic obstructive pulmonary disease (COPD), and benign prostatic hyperplasia (BPH). On physical examination, the patient was thin, appeared older than his age, and had mild abdominal distention, bilateral inguinal lymphadenopathy, and gynecomastia.

**Figure 1:**
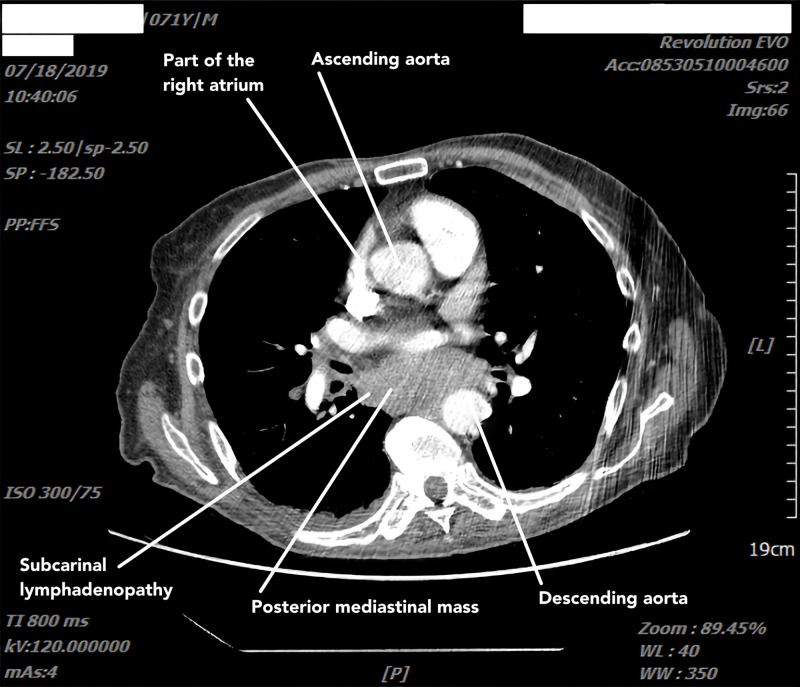
Chest CT scan with contrast showing posterior mediastinal mass and significantly enlarged lymph nodes in right and left paratracheal and subcarinal lymph nodes.

**Figure 2:**
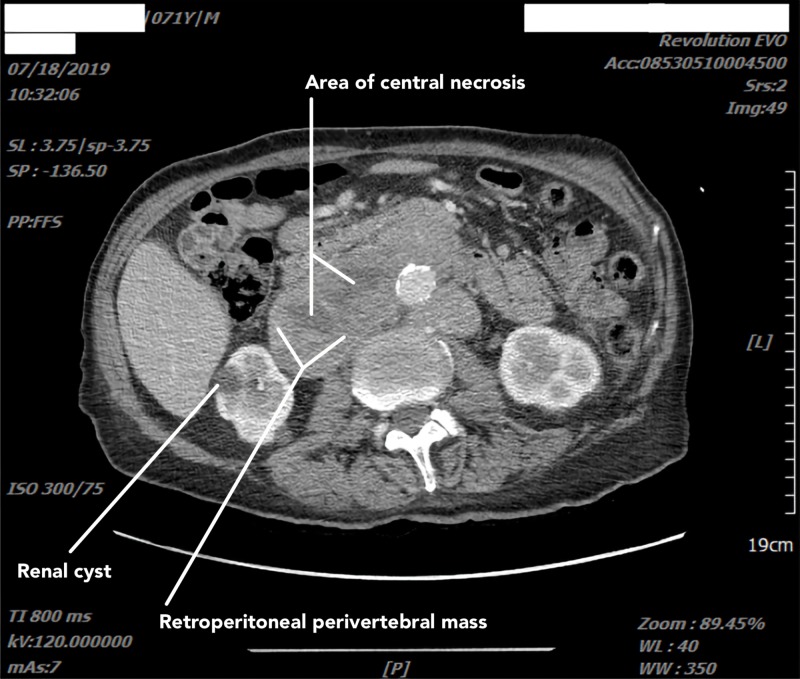
Abdominopelvic CT scan with contrast shows a retroperitoneal perivertebral mass measuring 11.9 cm in the largest dimension.

This constellation of symptoms necessitated an extensive workup. CT scan of the chest (Figure 1) revealed a posterior mediastinal mass and mediastinal lymphadenopathy (LAD). CT scan of the abdomen and pelvis (Figure 2) revealed large retroperitoneal masses measuring up to 11.9 cm with significant LAD, central hypo-density (necrosis) in bilateral iliac chains, and involvement of the perivertebral space. Multiple large inguinal lymph nodes with central necrosis were also seen. The above features with the history of KS and unilateral cryptorchidism were highly suggestive of germ cell tumors. Imaging showed no evidence of any anterior mediastinal masses, pulmonary metastasis, or liver metastasis, and a CT scan of the brain did not reveal any leptomeningeal metastasis.

Serum lab profile [including complete blood count (CBC), comprehensive metabolic panel (CMP), coagulation studies], fecal occult blood test (FOBT), and serum tumor markers [including alpha-fetoprotein (AFP), beta human chorionic gonadotropin (β-HCG), lactate dehydrogenase (LDH), and placental alkaline phosphatase (PLAP)] were positive only for mild anemia (Hct 34.1 & Hb 10.8) as well as elevated LDH level of 714. The remainder were negative or normal.

Colonoscopy with biopsy was performed, which did not reveal any malignant transformation or colorectal cancer.

**Figure 3:**
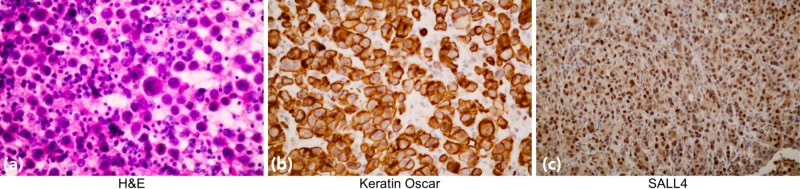
a) H&E staining demonstrating high-grade/poorly differentiated malignant neoplasm with necrosis, replacing most portions of the lymph node. b & c) Immunohistochemistry (IHC) studies reveal strong expression of keratin OSCAR, and SALL4.

The surgical team was consulted to perform an excisional biopsy of the inguinal lymph nodes. The resultant tissue samples revealed a poorly differentiated embryonal cell carcinoma. Morphologic review of the slides demonstrates high-grade/poorly differentiated malignant neoplasm with necrosis replacing most portions of the lymph node. Immunohistochemistry (IHC) studies revealed strong expression of tumor cells for keratin OSCAR, SALL4, and focal expression for keratin-7, along with a rare expression for OCT3/4. Tumor cells showed a lack of expression for CD30, PLAP, TTF1, CDX2, GATA-3, Calretinin, HCG, Inhibin, and SATB2 (Table 1 and Figure 3).

**Table 1:** Summary of gene expression in tumor tissue sample.

Immunohistochemistry (Antibody Name)	Result(s)
Calretinin (SP13), CDX2 (CDX2-88), GATA-3, TTF-1, SATB2	Negative
HCG, Inhibin	Negative
SALL4 (6E3)	Positive, strong expression
Keratin (OSCAR)	
Keratin 7 (OV-TL 12/30)	Positive, patchy
OCT 3/4 (N1NK)	Rare expression
Placental Alkaline Phosphatase (PLAP)	Negative
CD30 (JCM182)	

**Figure 4:**
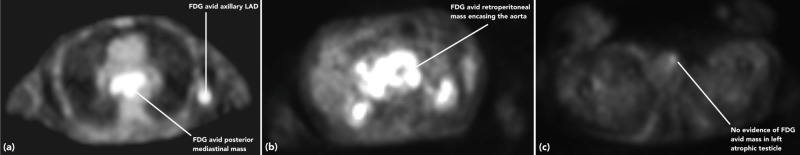
Positron emission tomography-computed tomography (PET-CT) demonstrating: a) extensive FDG-avid posterior mediastinal mass, b) retroperitoneal lymphadenopathy (LAD) encasing the aorta and likely displacing the inferior vena cava (IVC), c) with no evidence of tumor in the left atrophic testis.

Positron emission tomography-computed tomography (PET-CT) was subsequently obtained which revealed extensive FDG-avid LAD seen throughout the body (axillary, mediastinal, paraesophageal, retrocrural, mesenteric, inguinal) with the exception of the neck (Figure 4). There was extensive FDG-avid retroperitoneal LAD encasing the aorta and likely displacing the inferior vena cava (IVC). LAD was also noted to extend into the bilateral common and external iliac chains as well as to the right inguinal region.

Staging (2017 AJCC TNM Classification System for Testicular Cancer; Table 2): pTx: primary tumor cannot be assessed pN3: metastasis with a lymph node mass >5 cm in the greatest dimension M1b: distant metastases other than non-regional nodal or lung S2: LDH 1.5-10.0 × ULN or HCG 5,000-50,000 mIU/ml or AFP 1,000-10,000 ng/ml

Given the above parameters, an initial diagnosis of advanced stage IIIC testicular embryonal germ cell tumor was made (Table 2).

**Table 2:** a) 2017 AJCC TNM Classification System for Testicular Cancer, b) AJCC Prognostic Stage Grouping System for TGCTs.

2017 AJCC TNM Classification System for Testicular Cancer				
Tumor (T)	cTx	Primary tumor cannot be assessed		
	cT0	No evidence of primary tumors		
	cTis	Germ cell neoplasia in situ (GCNIS)^a^		
	cT4	Tumor invades scrotum with or without vascular/lymphatic invasion		
	pTx	Primary tumor cannot be assessed		
	pT0	No evidence of primary tumors		
	pTis	Germ cell neoplasia in situ (GCNIS)		
	pT1	pT1a	Tumor limited to testis (including rete testis invasion) without lymphovascular invasion	Tumor <3 cm in size^b^
		pT1b		Tumor ≥3 cm in size^b^
	pT2	Tumor limited to testis (including rete testis invasion) with lymphovascular invasion; or tumor invading hilar soft tissue or epididymis or penetrating visceral mesothelial layer covering the external surface of tunica albuginea with or without lymphovascular invasion		
	pT3	Tumor invades spermatic cord with or without lymphovascular invasion		
	pT3	Tumor invades scrotum with or without lymphovascular invasion		
Node (N)	pNx	Regional lymph nodes cannot be assessed		
	pN0	No regional lymph node metastasis		
	pN1	Metastasis with a lymph node mass ≤2 cm in greatest dimension and ≤5 nodes positive (none >2 cm in greatest dimension)		
	pN2	Metastasis with a lymph node mass >2 cm but not >5 cm in the greatest dimension; or >5 nodes positive (none >5 cm); or evidence of extranodal extension of tumor		
	pN3	Metastasis with a lymph node mass >5 cm in the greatest dimension		
Metastasis (M)	M0	No distant metastases		
	M1	M1a	Nonregional nodal or lung metastases	
		M1b	Distant metastases other than nonregional nodal or lung	
Serum markers (S)	Sx	Marker studies not available or not performed		
	S0	Marker study levels within normal limits		
	S1	LDH <1.5 × ULN and HCG <5,000 mIU/ml and AFP <1,000 ng/ml		
	S2	LDH 1.5–10.0 × ULN or HCG 5,000–50,000 mIU/ml or AFP 1,000–10,000 ng/ml		
	S3	LDH >10.0 × ULN or HCG >50,000 mIU/ml or AFP >10,000 ng/ml		
Note: ^a^Except for Tis confirmed by biopsy and T4, the extent of the primary tumor is classified by radical orchiectomy. Tx may be used for other categories for clinical staging. ^b^Subclassifications of pT1 apply to only pure seminoma				

AJCC Prognostic Stage Grouping System for TGCTs					
Stage		T	N	M	S
0		pTis	N0	M0	S0
I	I	pT1-4	N0	M0	Sx
	IA	pT1	N0	M0	S0
	IB	pT2-4	N0	M0	S0
	IS	Any pT/Tx	N0	M0	S1-3
II	II	Any pT/Tx	N1-3	M0	Sx
	IIA		N1	M0	S0
					S1
	IIB		N2	M0	S0
					S1
	IIC		N3	M0	S0
					S1
III	III		Any N	M1	S0
	IIIA		Any N	M1a	S1
					S0
	IIIB		N1-3	M0	S2
			Any N	M1a	
	IIIC		N1-3	M0	S3
			Any N	M1a	
				M1b	Any S

In light of advanced metastatic disease, chemotherapy was planned. A regimen of bleomycin, etoposide and platinum (BEP regimen) × 4 cycles of 21 days was ordered (Bleomycin 30U IV Weekly on days 1, 8 and 15; etoposide 100 mg/m² on day #1-5 day and cisplatin 20 mg/m² on day #1-5).

During cycle 1, day 15, the patient's treatment course was complicated by chemotherapy-induced severe neutropenia, with weakness, fatigue, diarrhea, and decreased breath sounds. As such, his day 15 dose was delayed and he was transferred to the ER and admitted. A complete infectious workup was initiated and he was started on empiric antibiotics as well as Neupogen 300 mcg SQ daily for five days. He reported significant improvement in symptoms and remained afebrile with improvement in his neutropenia throughout the remainder of his treatment course. Bleomycin was discontinued in the fourth cycle due to concerns regarding developing interstitial lung disease.

**Figure 5:**
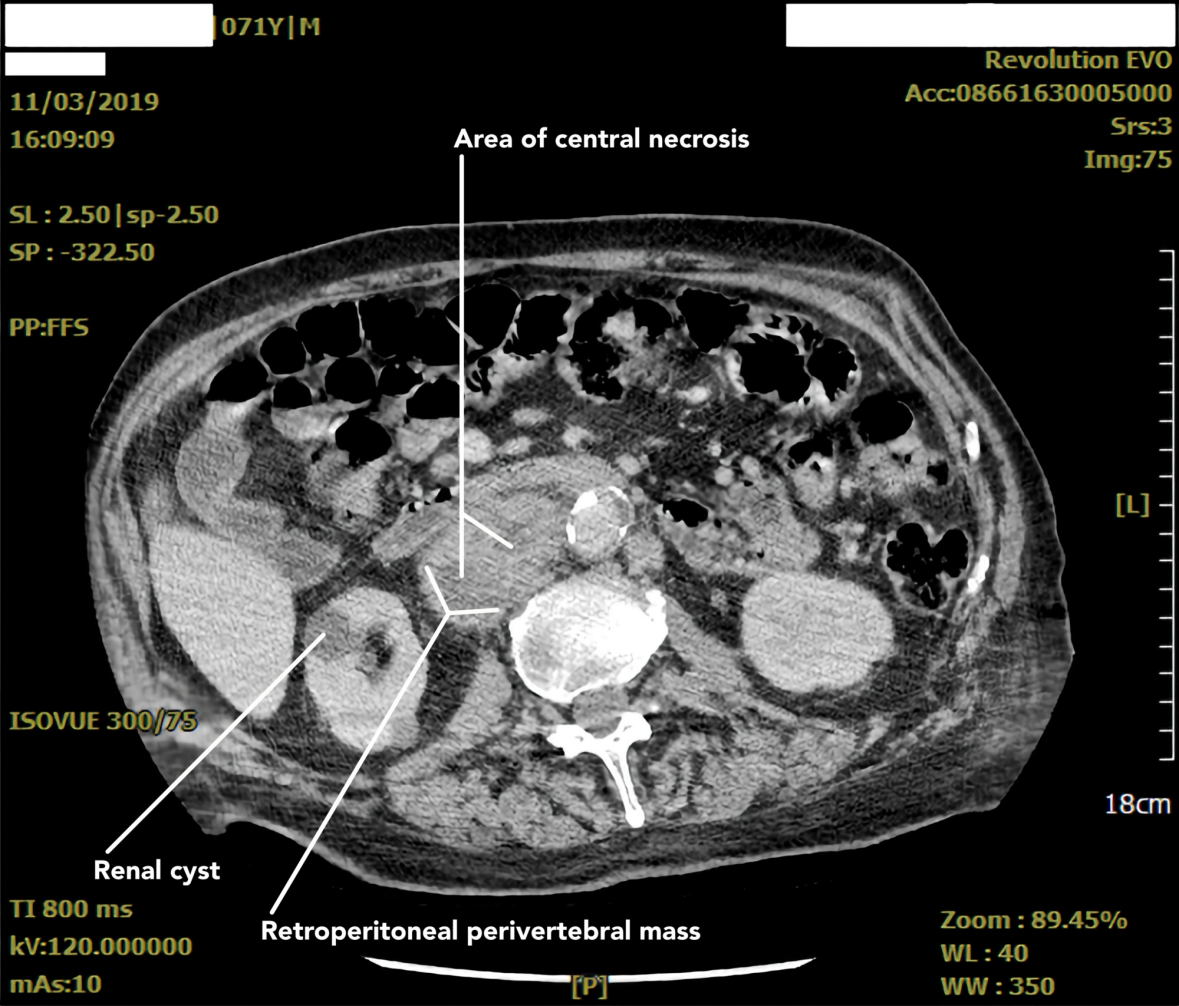
Abdominopelvic CT scan with contrast shows retroperitoneal masses including a heterogenous mass adjacent to the aorta to the right of the midline at the level of the kidneys measuring 11.9 cm in the largest dimension.

**Figure 6:**
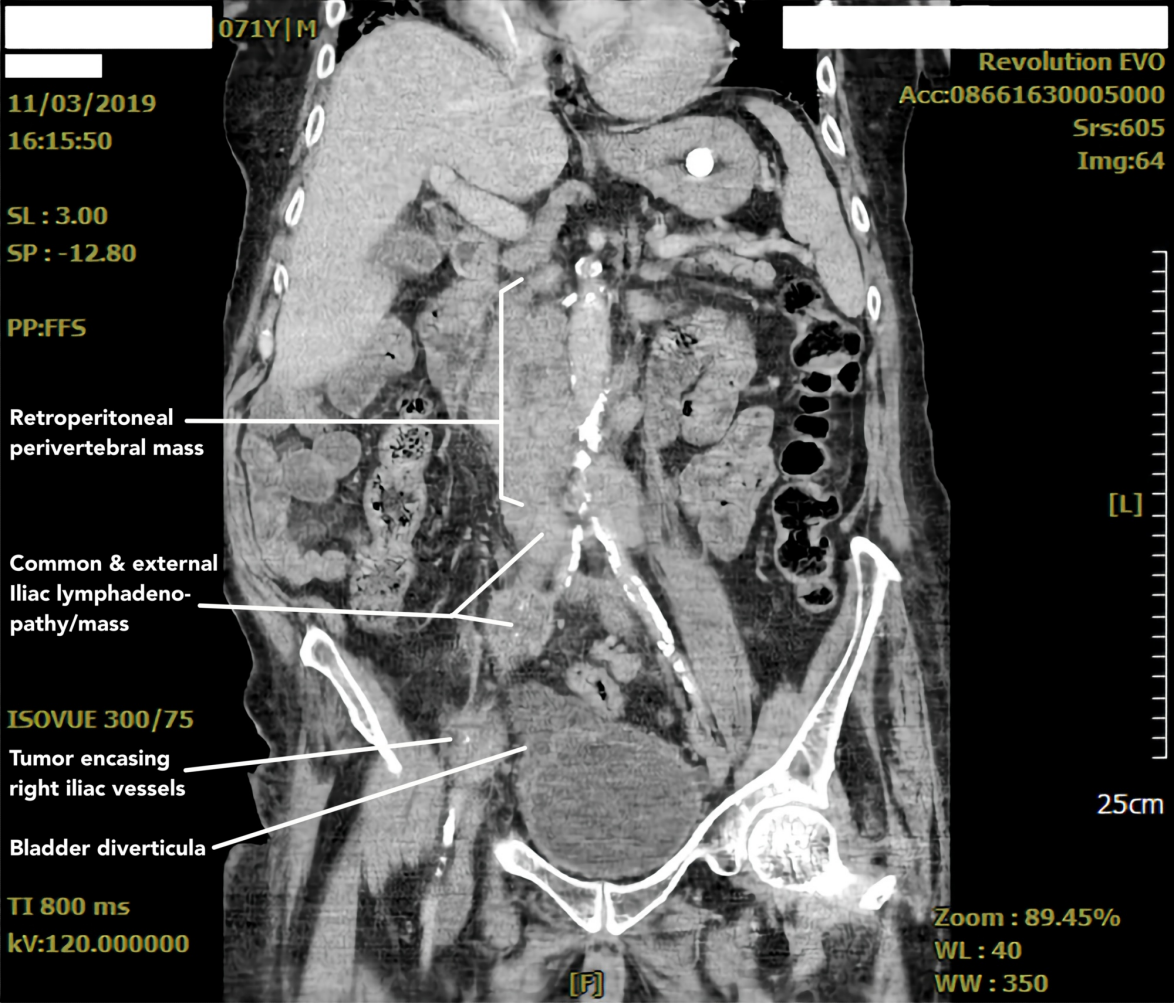
Abdominopelvic CT scan with contrast showing multiple large heterogenous retroperitoneal masses, similar in appearance compared to prior imaging. It also revealed redemonstration of scattered sub centimeter mesenteric lymphadenopathy.

After completing four cycles of chemotherapy, a repeat CT abdomen and pelvis was obtained (Figures 5, 6) in October 2019 to evaluate his response to treatment. It yielded redemonstration of multiple large retroperitoneal masses including a heterogeneous mass adjacent to the aorta to the right of midline at the level of the kidneys again measuring up to 11.9 cm. Additionally, it showed a 5.1 × 4.1 cm heterogenous mass just lateral to the right iliac artery abutting the right iliopsoas muscle. New findings included multiple mildly enlarged mesenteric lymph nodes measuring up to 1.3 cm. When compared to prior imaging, there was no significant decrease in the size of the masses or lymph nodes on follow-up imaging. Follow-up CT chest also revealed new bilateral diffuse ground-glass opacifications which were attributed to bleomycin toxicity.

The patient was readmitted in December 2019 for bilious vomiting and right leg pain. He was found to have a right superficial and common femoral vein deep vein thrombosis (DVT) attributed to compression from the right iliac tumor. Additionally a workup for small bowel obstruction necessitated surgical exploration. He was optimized and the surgical team planned to perform a diagnostic laparoscopy for the obstruction. Furthermore, a multi-disciplinary decision was made that the patient would benefit from tumor debulking if possible, considering he was non-responsive to chemotherapy.

**Figure 7:**
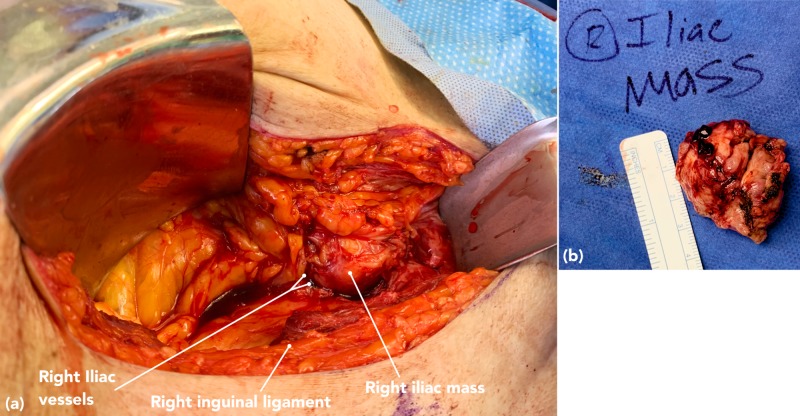
a) Right retroperitoneal exploration for right iliac tumor debulking revealed tumor encasing the right iliac vessels. b) Resected right iliac mass.

On laparoscopic exploration there were no findings that could explain a small bowel obstruction. A retroperitoneal dissection was begun for tumor debulking, and was eventually converted to an open right retroperitoneal dissection (Figure 7a). Although the tumor was noted to be encasing the right iliac vessels, we were successfully able to debulk this portion of tumor (Figure 7b). Additional tumor was encountered along the proximal right iliac vein and encasing the IVC, which was ultimately deemed unresectable. The repeat staining and immunohistochemistry of the right iliac tumor was entirely unchanged from the initial tumor biopsy.

## Discussion

**Figure 8:**
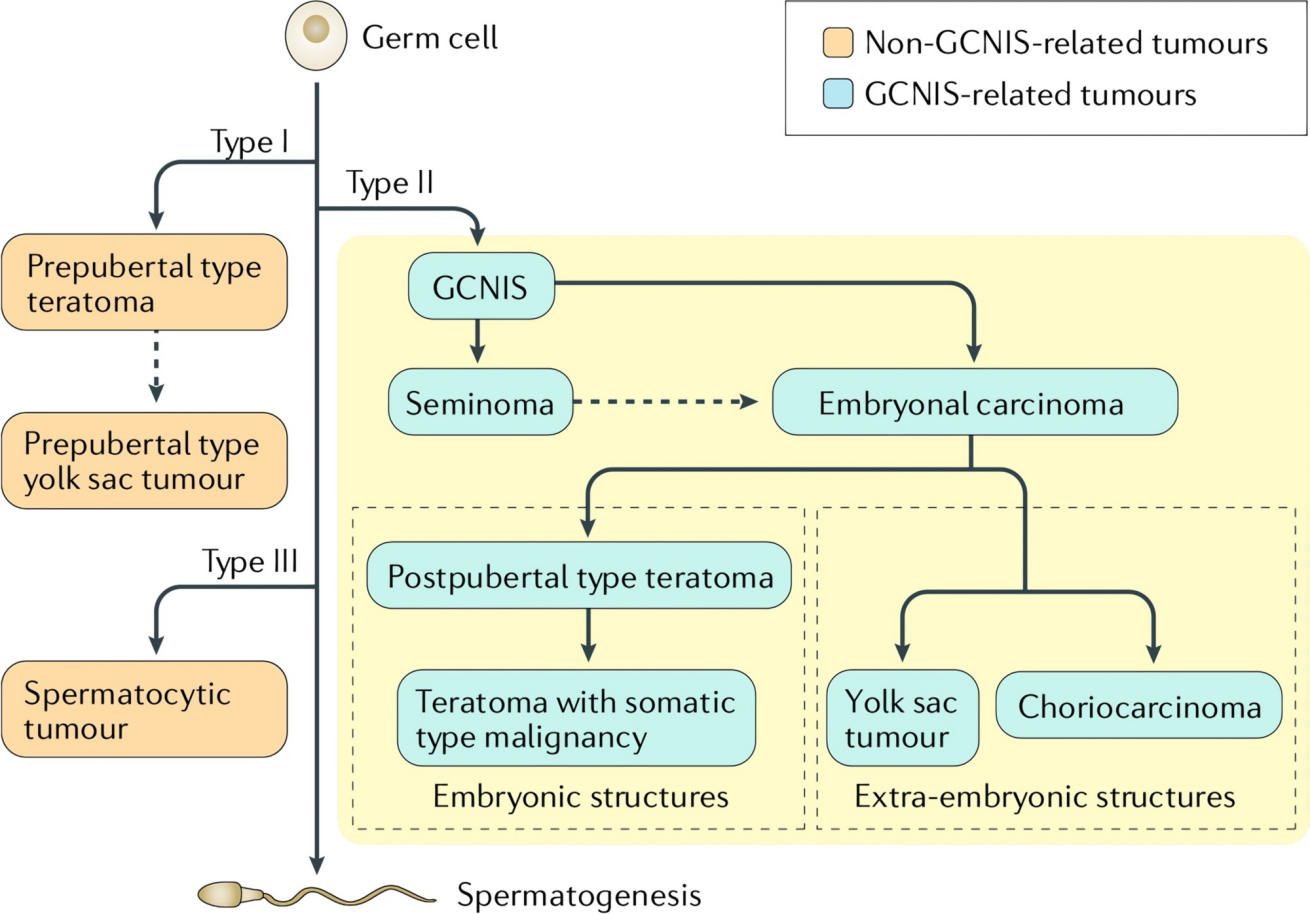
Schematic representation of the types of testicular germ cell tumors. Non-GCNIS-related germ cell tumors include prepubertal type teratomas and yolk sac tumors (also known as type I testicular germ cell tumors (TGCTs); yolk sac tumors can originate from teratomas) as well as spermatocytic tumors (also known as type III TGCTs). GCNIS: Germ cell neoplasia in situ Published with permission from [9].

Germ cell tumors (GCTs) traditionally were classified on the basis of their histological composition (i.e., H&E staining and Ab stained tumor sections). The World Health Organization (WHO) 2016 classification (summarized in Figure 8) of testicular germ cell tumors takes into account the germ cell involved, the embryonic and extra-embryonic lineages present, the histological composition of the tumor, the developmental potential of the cells of origin and their association with pathogenesis, and the age of onset of testicular cancer. Most GCT develops from a precancerous lesion called germ cell neoplasia in situ (GCNIS). Thus, the WHO classification defines the two major entities of TGCTs as GCNIS-related TGCTs and non-GCNIS-related TGCT [9]. This GCNIS is a precancerous lesion and is virtually always present together with the mature GCNIS-derived germ cell tumor when examined histologically. This lesion has a 50% chance of progressing to cancer within five years [9].

GCNIS mimics primordial/embryonic germ cells and expresses a defined set of embryonic mRNAs, miRNAs, and protein biomarkers, including the proteins mast/stem cell growth factor receptor KIT, placental alkaline phosphatase 1 (PLAP1, also known as ALPP), OCT4, NANOG, and the transcription factor SOX17 [10-11]. Each of these is informative as a diagnostic biomarker for GCNIS and seminoma, although their specificity and sensitivity differ.

Seminomas express SOX17 mRNA and/or protein, whereas embryonal carcinoma cells express SOX2 mRNA and/or protein [12]. The relevance of this transition during normal germ cell formation during embryogenesis has been elegantly demonstrated [13]. In fact, the transition from embryonic stem cells to primordial germ cells (PGCs) is driven by the switch from SOX2 to SOX17 expression.

**Figure 9:**
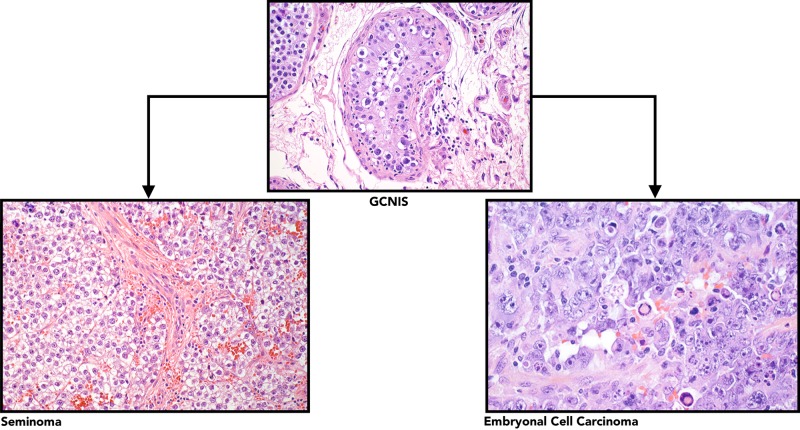
Histological composition of different TGCTs, as assessed by H&E staining under light microscopy. (Panel a; 400X) Germ cell neoplasia in situ (GCNIS) is the precursor lesion to Type II TGCTs. Histologically, these are seminomas (panel b; 200X) or nonseminomas such as embryonal carcinoma (panel c; 400X). TGCT: Testicular germ cell tumor; H&E: Hematoxylin and eosin Published with permission from [9].

Embryonal cell carcinoma usually affects young males, most of whom already have metastasis at the time of diagnosis. They share a few markers with seminomas, e.g., OCT 3/4 and PLAP [14]. They differ in their positivity for CD30 and cytokeratin. Also, they lack positivity for KIT (CD117). Hemorrhage and necrosis usually indicate a mixture with teratoma, choriocarcinoma, or both. A side-by-side comparison of seminomas and germ cell carcinomas are summarized in Table 3 and Figure 9.

**Table 3:** Literature review, comparison of seminoma and embryonal carcinoma based on microscopic features and immunohistochemistry (IHC) markers. *Please refer to Figure 9 for histologic comparison of these tumors.

TGCTs	Seminoma	Embryonal carcinoma
Micro*	Shows clear seminoma cells divided into poorly demarcated lobules by delicate septa. Microscopic examination also reveals large cells with distinct cell borders, pale nuclei, prominent nucleoli, and a sparse lymphocytic infiltrate.	Shows sheets of undifferentiated cells as well as primitive glandular differentiation. The nuclei are large and hyperchromatic. Histologically the cells grow in alveolar or tubular patterns, sometimes with papillary convolutions. More undifferentiated lesions may display sheets of cells. Well-formed glands are absent.
Immuno-histo- chemical markers	OCT 3/4, SALL4, PLAP1 (aka ALPP), NANOG	OCT 3/4, SALL4, PLAP1 (aka ALPP), NANOG
	Transcription factor SOX17	Transcription factor SOX2
	KIT (CD117) positive	KIT (CD117) negative
	Few scattered keratin-positive cells may also be present.	Cytokeratin positive
	CD30 negative	CD30 positive (highly sensitive & specific)

Two key issues in establishing the diagnosis (Table 4) of retroperitoneal (extragonadal) GCTs are the exclusion of metastasis from a primary testicular GCT and distinguishing an extragonadal nonseminomatous GCT from another type of poorly differentiated cancer.

**Table 4:** Differential diagnosis in our patient. GCT: Germ cell tumor; KS: Klinefelter syndrome; PET/CT: Positron emission tomography/computed tomography.

Differential diagnosis	Rules in	Rules out
Advanced Testicular GCT	History of KS with unilateral cryptorchidism	
	Posterior mediastinal and retroperitoneal mass on CT scan	
	Histopathology of inguinal lymph node biopsy with tissue markers	
	No evidence of tumor in the left atrophic testis on PET/CT scan	
Mediastinal GCT	History of KS	No evidence of tumor in the anterior mediastinum on PET/CT scan
	Histopathology of inguinal lymph node biopsy with tissue markers	
Other extragonadal GCTs	History of KS	History of cryptorchidism
	Histopathology of inguinal lymph node biopsy with tissue markers	Primary testicular germ cell tumor cannot be excluded
Lymphoma/leukemia	Age	No splenomegaly
	Widespread lymphadenopathy	LN biopsy and peripheral blood smear
	Hepatomegaly	CD30 negative (Hodgkin’s)

Testicular palpation is not sufficient to exclude a primary testicular GCT. Ultrasonography should be performed in all patients [15]. It may be difficult to distinguish true extragonadal GCTs from metastatic tumors in which the primary gonadal lesion has regressed [16-17].

Primary mediastinal GCTs occur in the anterior mediastinum, whereas testicular GCTs rarely metastasize to the anterior mediastinum (i.e., a GCT in the anterior mediastinum is unlikely to be a metastasis from a primary testicular GCT). Since our patient did not have any evidence of an anterior mediastinal mass, we were confidently able to rule out a mediastinal GCT. The presence of a posterior mediastinal mass on CT scan along with evidence of widespread metastasis (regional LAD), history of unilateral cryptorchidism, and embryonal cell carcinoma on histologic/IHC sampling supported the diagnosis of testicular (embryonal carcinoma) germ cell tumor located retroperitoneally.

An undescended testis in the retroperitoneal space during the embryonic period of human development may give rise to primary retroperitoneal testicular GCT in adulthood.

Globally, there is an increased level of tumor markers in 51% of cases of testicular tumors [18]. That being said, normal or nonelevated marker levels do not exclude the diagnosis of a GCT. About 90% of nonseminomatous tumors appear with an increase in one or two of the markers [19]. Our patient’s serum LDH concentration was elevated. LDH is considered a less specific marker, but its serum concentration is related to cancer volume. Its level may be elevated in 80% of subjects with advanced testicular cancer [20].

Embryonal cell carcinomas usually show strong expression of CD30 and OCT 3/4 (N1NK), but in our patient, CD30 was negative and OCT 3/4 showed a rare expression. Placental alkaline phosphatase (PLAP) is usually positive, but more patchy and weaker than in seminoma. In our patient’s tumor, it shows a complete lack of expression. These features contribute to the aberrancy of this cancer.

According to the International Germ Cell Cancer Collaborative Group (IGCCCG) prognostic grouping, this patient has a poor prognosis. Such patients should receive four cycles of BEP or etoposide, ifosfamide, and cisplatin (VIP). In an IGCCCG analysis, the five-year overall survival values of patients with intermediate prognosis and poor prognosis were 80% and 48%, respectively. Approximately 90% of patients with NSGCTs can achieve a complete remission with aggressive chemotherapy, and most can be cured [7-8]. Although our patient completed 4 cycles of BEP chemotherapy, there was no significant reduction in the size of his tumor or LAD. This may be explained by the aforementioned genetic aberrancy of this cancer.

A limitation in this case report includes the patient's one week delay within his first chemotherapy cycle (dose at day 15), as well as the discontinuation of bleomycin in his last cycle.

To summarize, our patient's case is exceedingly rare and difficult to diagnose in multiple respects: 1) Patients with KS usually develop extragonadal teratoma or mixed germ cell tumors, while ours was diagnosed with a testicular embryonal germ cell tumor. 2) GCTs usually develop in the testes while the remainder develops in the anterior mediastinum or retroperitoneum. This patient's tumor was present in the posterior mediastinum and retroperitoneum. 3) Patients with GCTs usually demonstrate elevated serum markers including LDH, AFP, and β-hCG. This patient only demonstrated an elevation in LDH. 4) GCTs usually show strong expression of CD30 and OCT3/4, and only show patchy staining of PLAP1. Our patient’s tumor tissue was negative for CD30, showed rare expression of OCT 3/4, and showed a complete lack of expression of PLAP. 5) Most patients with these tumors have a favorable response to chemotherapy, while this patient showed little to no response to the standard BEP regimen.

## Conclusions

Testicular nonseminomatous (embryonal) germ cell tumors are already a difficult disease entity to diagnose and treat. The uncommon presentation, including normal serum tumor markers, aberrant histological markers, and chemotherapeutic resistance made our case even more challenging to diagnose and treat.

Unfortunately, there are no specific guidelines when it comes to the management of KS patients with testicular GCTs with aberrant histological markers and normal serum tumor markers. As such, the selection of a chemotherapeutic agent must be done on a trial basis depending on the clinical status of the patient and the experience of the clinician. Of note, there are no data present in the literature supporting screening men with KS for testicular GCTs. This case report highlights a need for more specific modalities of diagnosis, treatment, and follow-up of this disease entity.
